# Building a middle-range theory of free public healthcare seeking in sub-Saharan Africa: a realist review

**DOI:** 10.1093/heapol/czx035

**Published:** 2017-05-16

**Authors:** Emilie Robert, Oumar Mallé Samb, Bruno Marchal, Valéry Ridde

**Affiliations:** 1 Research Institute of the McGill University Health Centre (RI-MUHC), Montréal, QC, Canada; 2 Division of Social and Transcultural Psychiatry, McGill University, Montréal, QC, Canada; 3 Equipe de recherche et d'intervention transculturelles (ERIT), CSSS de la Montagne, Montréal, QC; 4 Department of Health Sciences, Université du Québec en Abitibi-Témiscamingue, QC, Canada; 5 Institute of Tropical Medicine of Antwerp, Health Services Management Unit, Antwerp, Belgium; 6 School of public health (ESPUM), Montreal University, Montréal, QC, Canada and; 7 University of Montreal Public Health Research Institute (IRSPUM), Montréal, QC, Canada

**Keywords:** Access to healthcare, health policy and systems research, health user fees, middle-range theory, realist review, sub-Saharan Africa

## Abstract

Realist reviews are a new form of knowledge synthesis aimed at providing middle-range theories (MRTs) that specify how interventions work, for which populations, and under what circumstances. This approach opens the ‘black box’ of an intervention by showing how it triggers mechanisms in specific contexts to produce outcomes. We conducted a realist review of health user fee exemption policies (UFEPs) in sub-Saharan Africa (SSA). This article presents how we developed both the intervention theory (IT) of UFEPs and a MRT of free public healthcare seeking in SSA, building on Sen’s capability approach. Over the course of this iterative process, we explored theoretical writings on healthcare access, services use, and healthcare seeking behaviour. We also analysed empirical studies on UFEPs and healthcare access in free care contexts. According to the IT, free care at the point of delivery is a resource allowing users to make choices about their use of public healthcare services, choices previously not generally available to them. Users’ ability to choose to seek free care is influenced by structural, local, and individual conversion factors. We tested this IT on 69 empirical studies selected on the basis of their scientific rigor and relevance to the theory. From that analysis, we formulated a MRT on seeking free public healthcare in SSA. It highlights three key mechanisms in users’ choice to seek free public healthcare: trust, risk awareness and acceptability. Contextual elements that influence both users’ ability and choice to seek free care include: availability of and control over resources at the individual level; characteristics of users’ and providers’ communities at the local level; and health system organization, governance and policies at the structural level.

Key Messages

- Using a realist review, we developed both the intervention theory of user fee exemption policies (UFEPs) and a middle-range theory of free public healthcare seeking in sub-Saharan Africa.
- UFEPs place an additional resource in people’s wallets: free healthcare at the point of delivery. This resource gives them the opportunity to use public health services whenever they feel the need, without being dissuaded by cost.
- Three mechanisms explain why beneficiaries would choose to seek free public healthcare: trust, risk awareness, and acceptability.
- Individual, local, and structural conversion factors influence users’ ability and choice to seek free care. They constantly interact with and shape users’ capability space.

## Introduction

Healthcare access in low- and middle-income countries (LMICs) is a constant concern of governments and global health actors. Following the 1978 Declaration of Alma-Ata, primary healthcare was supposed to be ‘made universally accessible’ (World Health Organization and United Nations Children’s Fund, 1978). The focus on universal health coverage in the Sustainable Development Goals shows this issue remains a major challenge. User fee exemption policies (UFEPs), frequently cited as options to improve healthcare access, are being implemented in several LMICs, notably in sub-Saharan Africa (SSA) (Robert *et al.* 2012 ; Richard *et al.* 2013). We undertook a realist review to answer three research questions: What are the outcomes of UFEPs implemented in SSA? Why do they produce such outcomes? What contextual elements come into play? (Robert *et al.* 2012

Realist reviews are a new form of knowledge synthesis that opens the ‘black box’ of an intervention by showing how it triggers mechanisms in specific contexts to produce outcomes. Their aim is to produce middle-range theories (MRTs) that specify how interventions work, for which populations, and under what circumstances (Pawson *et al.* 2005; Pawson 2006). MRTs (Merton 1968) are considered a suitable level of abstraction to maintain the operationality needed for applied research while producing cross-cutting lessons (Weick 1989; Hercot *et al.* 2011), especially on access to care (Dixon-Woods *et al.* 2006).

Because the realist review is an emerging method, greater efforts are needed to report precisely and explicitly not only on the knowledge synthesis process, but also—and perhaps more importantly—on the theory-building process (Tricco *et al.* 2016). In this article we present the intervention theory (IT) of UFEPs, which is a theoretical explanation of how they are supposed to produce their intended outcomes, and propose an MRT of free public healthcare seeking in SSA; and report how we built both theories.

## Methods

Methodological details for this realist review have been published elsewhere (Robert *et al.* 2012. Here we present the study context and briefly explain the realist approach principles. We then describe the review process, from specifying the problem and the research focus, to building the IT, and then the MRT. We report this process retrospectively, to convey its complexity and iterative nature, as well as the reflexivity that guided us.

### Study context

In SSA, user fees are a major barrier to healthcare access (Gilson and McIntyre 2005; Rutherford *et al.* 2010). As 70% of the population were living on less than US $2 per day in 2010 (World Bank 2014), the specter of the medical poverty trap is unremitting (Whitehead *et al.* 2001). In this context, removing user fees seems an expeditious solution to improve access. According to a simulation (James *et al.* 2005), it would prevent the deaths of 153 000–305 000 children under 5 every year in 20 African countries. Some also see it as moving toward universal health coverage (Yates 2009; Ridde and Queuille 2010; Meessen 2013). Many SSA countries have therefore introduced UFEPs for specific categories of population or health services.

Numerous empirical studies on UFEPs have reported heterogeneous and sometimes contradictory results. Their designs have suffered from methodological weaknesses (Lagarde and Palmer 2011) associated with real-life UFEP implementation contexts (Ridde and Haddad 2013), which limited the use of traditional systematic reviews. We therefore designed a realist review (Pawson *et al.* 2005) to explain the diversity of UFEP outcomes (Robert *et al.* 2012.

Our review focuses on SSA countries for three reasons. First, their health indicators are among the weakest and their financial barriers to care the most flagrant. Second, their contexts are relatively comparable, even with cultural and socio-economic differences, and as a group they differ significantly from Asia, Latin American and North Africa, in terms of health resources, population wealth, and health systems organization. Finally, as researchers we are familiar with this region and better able to understand the ramifications of empirical studies in these contexts.

### The realist approach

Pawson and Tilley (1997) proposed that complex social interventions should be evaluated according to principles of realism. According to these principles, an intervention does not work per se; rather, it is the actors who make the intended outcomes of an intervention possible or not through various mechanisms. These include their reasoning, attitudes, and behaviours (Lacouture *et al.* 2015), all influenced by contexts in which the intervention is implemented. Outcomes are thus the product of interaction between mechanisms and contexts.

Realist reviews are a form of knowledge synthesis based on those principles (Pawson 2006). Building on iterative analysis of theoretical and empirical literature, realist reviews start with ITs and produce MRTs showing the interactions among an intervention’s context, mechanisms, and outcomes. Guided by the theoretical literature, realist reviews search the empirical literature for tendencies, or demi-regularities, in interactions among these elements. These interactions take the form of context–mechanism–outcome (CMO) configurations. Constructing an MRT involves identifying demi-regularities, supported by theoretical assumptions. An MRT must have the level of abstraction needed to explain the diversity of outcomes produced by an intervention in different contexts (Pawson 2000).

### The review process

#### Specifying the problem under investigation and the research focus

We began by immersing ourselves in data and literature on UFEPs. Given the abundance of material, we narrowed down the types of UFEPs to focus on the recent regional or national government-mandated UFEPs implemented in SSA countries that provided free healthcare at the point of delivery in a universal way (Carey and Crammond 2014), regardless of diagnosis or type of illness. These UFEPs either made services entirely free for specific categories of populations or made certain essential, primary or basic services free for everyone. They differ from exemptions targeting the poor and exemptions for specific conditions or treatments (e.g. free treatment for Buruli ulcer or leprosy, vaccination, prenatal consultations), which are not part of the review. Policies establishing free caesarean section, notably in Mali and Senegal, have also been excluded because the caesarean section is a medical procedure decided by the healthcare staff. Empirical studies of UFEPs were identified through a literature search strategy (Robert *et al.* 2012. Inclusion and exclusion criteria were applied.

**Figure 1.**
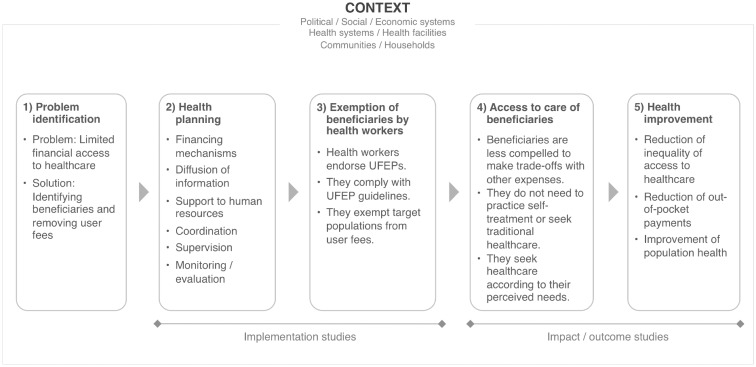
Generic process theory of UFEPs

We also conducted two modelling exercises: one to clarify the problems targeted by UFEPs (Supplementary Figure S1) and the other to illustrate UFEP generic process theory, displaying the processes leading to intended outcomes (Weiss 1997) (Figure 1). Preliminary versions were discussed with key informants, including policy makers and health managers, during 15 interviews in Mali and Burkina Faso and a focus group in Burkina Faso. These reflections led us to move away from the technical specifics of each UFEP and focus exclusively on their common feature, the partial lifting of the financial barrier.

According to UFEP generic process theory, health planning involves implementing a set of strategies—funding arrangements, information campaigns, coordination, monitoring, evaluation measures etc.—that enable health workers to deliver free care. To the extent that they endorse these policies, health workers comply with UFEPs, exempting target populations from user fees. Process evaluations and policy implementation studies focus on this process. UFEPs foster access for selected populations, as measured by increased use of free services. Ultimately, they should contribute to improving population health, reducing healthcare access inequities and out-of pocket payments. These outcomes are measured by impact studies or economic evaluations.

In our modelling of the UFEP generic process theory, providers and users emerged as the two main actors whose agency affects UFEPs. We began by considering both, exploring theoretical writings on health services organization and healthcare access. This exploratory process did not involve systematic analysis of the empirical studies. Faced with the complexity of a dual analysis calling for different theoretical frameworks, we narrowed the scope of our realist review to focus on users’ experience. During this process, a more precise research question took shape: How do UFEPs enhance use of public healthcare services? This was then framed as two sub-questions: in what contexts do UFEPs facilitate use and through what mechanisms?

#### Building the IT of UFEPs

Having decided to focus on users’ experience, we assessed the empirical studies found through the literature search, in terms of relevance to the research question and scientific rigor. We first determined whether they contained information to inform the theory-building process, applying the principle of conceptual saturation (Thomas and Harden 2008). We then used the Mixed Method Appraisal Tool (Pluye *et al.* 2011) to assess scientific rigor (Robert 2015). Data were extracted using NVivo9 software, or manually for documents that could not be uploaded. Adopting an integrated approach for the analysis (Bradley *et al.* 2007), we developed a coding tree based on the concepts of the realist approach (CMO), which we continuously adjusted and fine-tuned to incorporate relationships emerging from studies and concepts from theoretical literature.

The theoretical literature included traditional and recent conceptual frameworks and models of healthcare access or use, as well as those concerning LMICs. We examined recent knowledge syntheses on healthcare access (see Supplementary Material S1) and explored theoretical writings from related disciplines such as development studies, anthropology, and health economics in an *ad hoc* way as new research avenues were opened by our reflections. We did not examine these writings in-depth; rather, we read them through the realist lens, sometimes repeatedly. We searched for theories or concepts that echoed realist concepts while providing hypotheses to explain UFEP outcomes in empirical studies. From this iterative analysis of empirical and theoretical literature, we accumulated knowledge that contributed to the IT construction.

**Figure 2.**
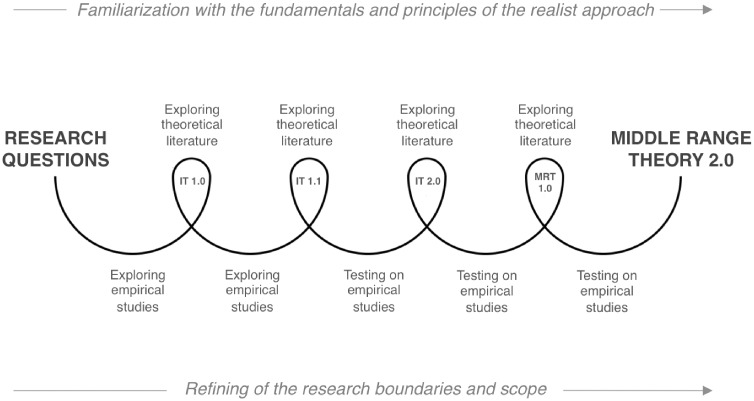
Iterative research process of the realist review

Two provisional ITs (IT 1.0; IT 1.1) were tested on empirical studies and the analyses fed back into our reflection in a process of induction by elimination (Deslauriers 1997). Once we had found the most plausible mechanism and underlying theory, we proposed a new IT (IT 2.0). Our analysis of the empirical literature confirmed its explanatory power to answer the research question. This version became the new starting point for analysis (Figure 2). Once coding was completed, the entire corpus was reviewed to standardize the coding tree and check consistency of interpretation (Thomas and Harden 2008).

During this step, we identified demi-regularities to highlight contexts in which the IT mechanism was triggered—or not—and the resulting outcomes. We adopted the retroductive approach, a process of retrospective reasoning by which the researcher moves from describing a phenomenon to what produces this phenomenon and the structures in which it is embedded (Bhaskar 1986, cited by Wai-chung Yeung 1997), using the concept of mechanism (McEvoy and Richard 2006).

The IT was thus built in the form of a puzzle whose pieces were the CMO configurations identified in the empirical literature. Configurations were often incomplete, as the mechanism was generally implicit. Missing links were inferred from the theoretical literature, which provided keys to explain relationships between context and outcome, using reasoning based on two questions: How can the mechanism be explained in this context and considering this outcome? Is this interpretation supported by theory? This inference was the first level of abstraction. It became a demi-regularity when the configuration appeared repeatedly. Similarly, any configuration identified only once but whose interpretation was strongly supported by theory was retained if it helped provide a clearer picture. Each demi-regularity thus contributed one or more puzzle pieces for a more complete and precise picture of the phenomenon. This dialogue between theoretical and empirical literatures helped us to iteratively develop, test and refine the IT.

#### The shift toward a MRT of free public healthcare seeking

In developing and testing the IT, we looked more closely at contexts in which UFEPs could trigger the intended mechanism. We encountered several examples in the empirical studies where expected UFEPs outcomes, such as increased health services use, did not result entirely from the expected mechanism. Rather, the contexts triggered other mechanisms leading to the same outcome. This led us to examine the complex phenomenon of healthcare seeking.

Our research focus thus shifted from UFEPs as the core intervention of our realist review to the complex phenomenon of free public healthcare seeking. To account for these mechanisms and contexts, we moved from the IT of exemption policies to the MRT of free public healthcare seeking (MRT 1). The following questions guided our new analysis: What other mechanisms lead to free public healthcare seeking? In what contexts are they triggered or hindered? And how do contexts and mechanisms interact?

When an empirical study provided evidence related to choices other than free healthcare seeking, such as using private or traditional care, the evidence was brought into the MRT as a counter-example of hindering contexts and their interaction with the mechanisms in users’ choice to seek (or not) free healthcare. Because free public healthcare seeking was the only outcome we examined systematically, these counter-examples do not explicitly appear in the MRT.

The process was complete when the final MRT (MRT 2) was able to explain the demi-regularities identified in the empirical studies. The same theory-building process and empirical materials were used to build, test and refine both the IT and the MRT. Figure 2 illustrates the continuous feedback loop between the theoretical and empirical literature.

## Results

In this section, we describe the IT and define the concepts. Then, we present demi-regularities emerging from the analysis of empirical studies as CMO configurations. A synthesis of the empirical literature can be found in the first author’s doctoral dissertation (Robert 2015). Finally, we propose an MRT on free public healthcare seeking in SSA.

### Contributions from the theoretical literature: empowerment as the mechanism triggered by exemption policies

UFEPs are intended to improve access, manifested as increased use of free health services. UFEPs are not easily classified into the common categories of policy instruments for behavioural change (Vedung 1998): they offer no tangible rewards (‘carrot’), are not coercive (‘stick’) and are not intended to inform or blame (‘sermon’). UFEPs offer users a service they may choose to use or not, with no direct financial pressure. Given this, the many conceptualizations of access to care seemed to us reductionist, being generally descriptive and focused on factors that facilitate or inhibit use. They were also relatively static (Ricketts and Goldsmith 2005), as they rarely conceptualize users as actors and do not consider their lived experience.

Exceptions are the models of Levesque *et al.* (2013), who see access as an opportunity, and of Thiede (2005), who defines access as ‘freedom to use’. More precisely, ‘freedom to use describes the social possibility and the individual ability to give direction to one’s will to use health services’ (Thiede 2005, p. 1453). This definition concurs with Sen’s work (1985), furthered by Kabeer (1999), on empowerment and capabilities. According to Currie and Wiesenberg (2003), a decision to seek healthcare requires that a set of choices be available to users; otherwise no decision is possible. Kabeer (1999) sees empowerment as a process in which an individual’s ability to make strategic life choices increases. This ability to choose is expressed in terms of resources (material, human, or social) and agency, i.e. the ability to make one’s own decisions and pursue one’s goals. Capabilities are the sum of resources and agency (Kabeer 1999).

According to these theoretical assumptions, free healthcare is an intangible resource that empowers users. With this new resource, it is assumed users will not only be less compelled to make trade-offs with other expenses or develop resource mobilization strategies, but will also be more likely to opt for public health services when they feel the need. However, users’ ability to take up this resource, i.e. to choose to use free health services, is influenced by conversion factors that are personal, local and structural (Robeyns 2005; Frediani 2010).

‘Individual factors’ are associated with one’s individual capacities, which could be physical conditions, levels of literacy, and so forth. […] ‘Local factors’ can be associated with facilities and collective norms. […] Last but not least are the ‘structural factors’ shaping the capability space. Market mechanisms and the political structure are examples of some of the underlying structural processes that affect people’s freedoms. (Frediani 2010, p. 178)

With regard to healthcare access, examples are: social status and income as personal factors; providers’ attitudes and practices as local factors; and public policies and health system governance as structural factors. These are interrelated and influence each other. The set of choices, abilities, and resources is the capability space (Frediani 2010). In a realist view, conversion factors may be understood as contextual factors contributing to or limiting users’ empowerment. Empowerment is the mechanism triggered by UFEPs. When triggered, it allows users to choose to use free public health services according to their needs.

**Figure 3.**
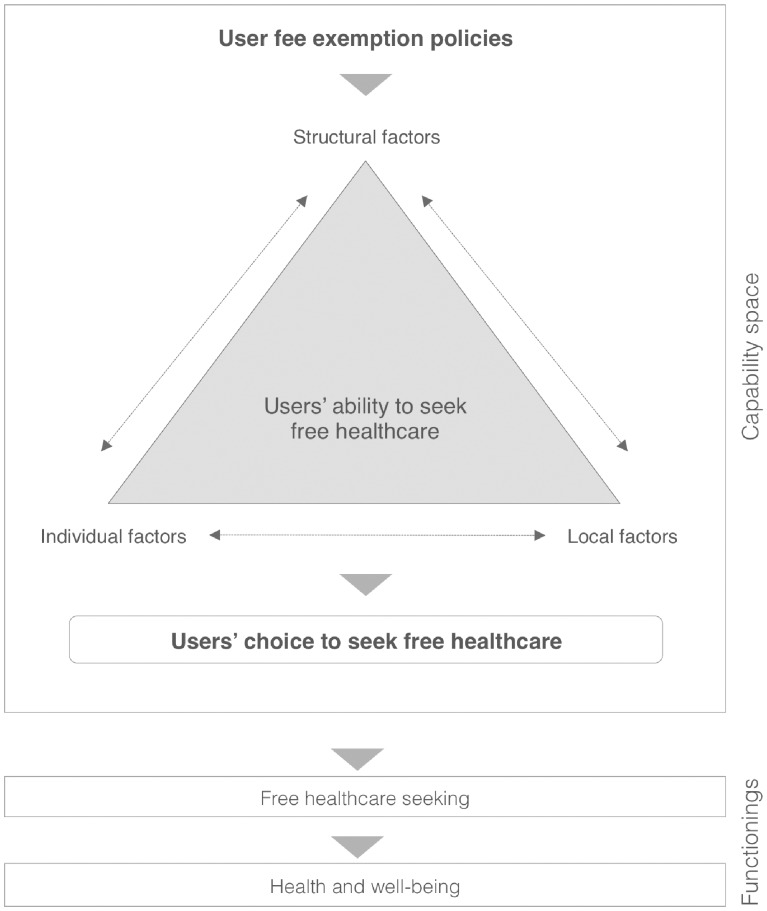
A capability approach to seeking free healthcare (IT)

We adapted Frediani’s (2010) capability approach to illustrate the free healthcare seeking phenomenon (Figure 3). According to this approach, functionings are what people value and what determine their choices (Frediani 2010). In care seeking, users pursue health and well-being. In realist terms, these functionings are the outcomes in the CMO configuration.

Drawing on these concepts, we proposed the following IT: by making public healthcare free, UFEPs empower users with an additional resource, enabling them to make strategic choices according to their needs (‘mechanism’). This contributes to improve healthcare access (‘outcome’). Users’ ability to choose to seek free care is also influenced by structural, local and individual factors (‘context’**)**.

Since mechanisms are usually invisible (Pawson 2013) and must be deduced through empirical studies, we considered users’ empowerment to have been triggered when indicators measuring use of services associated with UFEPs had improved. Two other outcomes were possible: not seeking care (or delaying/abandoning care seeking) and seeking informal and/or private healthcare. Berthet *et al.* (2009) suggest these may occur when: (i) the available resource is insufficient or not suited to needs; (ii) users do not have the capacity to take up the available resource, indicating that their capability space is constrained; or (iii) users choose not to take up this resource, regardless of its availability.

### Demi-regularities from empirical studies

We tested the IT on 69 empirical studies concerning 10 SSA countries. Robert’s doctoral dissertation (2015) explains how those studies were selected and their relevance and scientific rigor assessed. It also synthesizes the evidence from which the demi-regularities in Table 1 were drawn.

**Table 1.** Demi-regularities emerging from empirical studies

Theme	CMO configuration	Evidence
**Demi-regularity Type 1**		
**Contexts triggering or inhibiting users’ empowerment**		
Structural factors	If public healthcare and medicines are actually free of charge at the point of delivery for targeted users, and they are informed about it, then users’ ability to choose to seek free public health care according to their need is strengthened. This improves their access to care. (*DR 1-1*)	Ghana (Mills et al. 2008; Dzakpasu et al. 2012) Niger (Lagarde et al. 2012) South Africa (Wilkinson et al. 2001) Uganda (Deininger and Mpuga 2005; Nabyonga-Orem et al. 2011) Zambia (Masiye et al. 2010)
	If UFEP implementation is deficient and does not ensure actual free care and drugs, then users’ ability to choose to seek free public healthcare according to their need is not strengthened. This limits their access to care. (*DR 1-2*)	Uganda (Twikirize and O’Brien 2012) Tanzania (Kahabuka et al. 2012) Zambia (Hadley 2011; Carasso et al. 2012)
		
Geographic local factors	If health facilities are nearby, or if means of transportation are available, then users’ ability to choose to seek free public healthcare according to their need is strengthened. This improves their access to care. (*DR 1-3*)	Ghana (Mills et al. 2008) South Africa (Goudge et al. 2009b) Uganda (Nabyonga et al. 2005)
	If users are in a situation of geographic vulnerability, both regarding proximity of health facilities and availability of means of transportation, then their ability to choose to seek free public healthcare according to their need is limited. This prompts them to resort to informal or private health service providers when more easily accessible, to adopt a waiting position with respect to the evolution of their medical condition, or to forgo health care. (*DR 1-4*)	Senegal (Ministère de la santé et de la prévention médicale 2007) Sierra Leone (Diaz et al. 2013) South Africa (Goudge et al. 2009a) Tanzania (Pfeiffer and Mwaipopo 2013) Uganda (Deininger and Mpuga 2005; Kiguli et al. 2009; Rutebemberwa et al. 2009) Zambia (Hadley 2011)
		
Individual factor—financial resources	If users have limited financial resources and are unable to pay the indirect costs associated with accessing free public health services, including those related to transportation, then their ability to choose to seek free public healthcare according to their need is limited. This incites them to resort to providers who are more readily available, to forgo health care, or to adopt a waiting position with respect to the evolution of their medical condition. (*DR 1-5*)	Sierra Leone (Diaz et al. 2013) South Africa (Goudge et al. 2009b) Tanzania (Magoma et al. 2010) Uganda (Kiguli et al. 2009; Rutebemberwa et al. 2009) Zambia (Hadley 2011)
	If users have the financial resources that allow them to seek care according to their needs, then their perceptions of the quality of care influence their choice of providers. (*DR 1-6*)	Uganda (Pariyo et al. 2009) South Africa (Knight and Maharaj 2009)
		
Individual factor—social network	If users have a social network within the community that allows them to gain access to financial and/or material resources, then their ability to choose to seek free public healthcare according to their need is strengthened. This improves their access to care. (*DR 1-7*)	Niger (Diarra 2011) South Africa (Goudge et al. 2009b) Tanzania (Mrisho et al. 2007)
	If users have a social network within the health facilities, then patronage – support of a user by a known health service provider – allows them to benefit from actual free healthcare. (*DR 1-8*)	Niger (Ousseini 2010)
		
Local factors—social and cultural norms and beliefs	In the case of childbirth, the fact that the decision on the place of delivery lies in the hands of men as heads of the household limits women’s ability to choose to use free assisted delivery. When the head of the household owns the financial resources, persistent indirect costs further limit women’s ability to choose. (*DR 1-9*)	Ghana (Mills et al. 2008) Senegal (Witter et al. 2009) Tanzania (Mrisho et al. 2007; Mpembeni et al. 2007; Magoma et al. 2010)
	Where there is a discrepancy between sociocultural norms and beliefs, on one hand, and the supply of free health services on the other, then users’ ability to choose to seek free public healthcare according to their need is limited. This encourages domestic or traditional care practices. (*DR 1-10*)	Senegal (Ministère de la santé et de la prévention médicale 2007; Witter et al. 2009) Sierra Leone (Diaz et al. 2013) South Africa (Goudge et al. 2009b) Tanzania (Mrisho et al. 2007; Magoma et al. 2010; Pfeiffer and Mwaipopo 2013)
	When pregnant women perceive a lack of privacy in relation to assisted delivery by a man, then embarrassment diverts them from choosing free assisted delivery and leads them to favour home birth. (*DR 1-11*)	Senegal (Ministère de la santé et de la prévention médicale 2007; Witter et al. 2009) Tanzania (Mrisho et al. 2007)
**Demi-regularity Type 2**		
**Mechanisms triggered in the healthcare seeking choice-making process in a free care context**		
Trust	If providers demonstrate professionalism and empathy and meet users’ expectations, then users develop a sense of trust that encourages them to choose to use free public health services (and vice versa). (*DR 2-1*)	Ghana (Agyepong and Nagai 2010; Witter et al. 2013) Niger (Diarra 2011) Sierra Leone (Amnesty International 2011) Tanzania (Mrisho et al. 2007; Magoma et al. 2010; Kruk et al. 2008, 2009, 2010; Kahabuka et al. 2011, 2012) Uganda (Kajula et al. 2004; Rutebemberwa et al. 2009)
	UFEP implementation failures and pre-existing dysfunctional public health systems undermine relations between users and providers. They contribute to the emergence among users of a sense of distrust toward health service providers or the health system. This reinforces the bypass phenomenon or the choice of private providers or domestic care. (*DR 2-2*)	Ghana (Witter et al. 2007) Uganda (Twikirize and O’Brien 2012) Senegal (Ministère de la santé et de la prévention médicale 2007) Sierra Leone (Amnesty International 2011) Tanzania (Kruk et al. 2009; Kahabuka et al. 2012)
	If providers inflict humiliation on users who are in a situation of poverty, this contributes to users’ self-exclusion from free public healthcare. (*DR 2-3*)	Uganda (Kiguli et al. 2009) Zambia (Hadley 2011)
		
Risk awareness	If caregivers do not raise pregnant women’s awareness of the risks associated with childbirth, and if cultural norms value home birth, then pregnant women do not recognize the risks associated with this event and tend to opt for home birth. (*DR 2-4*)	Ghana (Mills et al. 2008) Tanzania (Mpembeni et al. 2007; Magoma et al. 2010)

Two types of demi-regularities emerged from the empirical literature. The first (Type 1) shows the influence of various contexts on users’ empowerment. These demi-regularities illuminate the key role of UFEP implementation and information in the care seeking process, both being structural preconditions for fostering empowerment. They also highlight local and individual factors that either constrain users’ capability space or strengthen it by facilitating their choice to seek free care. The second (Type 2) shows how users’ choice to use free public healthcare is shaped by their trust in providers and awareness of risks associated with their medical conditions. These demi-regularities reveal the importance of social and cultural factors.

Table 1 presents, the evidence used to infer logical links among CMOs, as manifested in the demi-regularities. It does not present all the studies reviewed, only those providing a puzzle piece. Other studies shed light on context and outcomes, and were helpful for addressing the general research question without contributing explicitly or empirically to demi-regularities. All 69 studies are referenced in Supplementary Material S2.

### A MRT of free public healthcare seeking

According to our MRT, UFEP beneficiaries would choose to seek free public healthcare when:

1. They trust the providers, the health facility, and more broadly the health system (mechanism: trust);
2. They perceive and are aware of risks associated with their medical condition, disease or pregnancy (mechanism: risk awareness); and
3. They perceive the choice to seek free healthcare as acceptable (mechanism: acceptability).

Trust here is ‘a state of mind in which the individual expects the person with whom she interacts to react in a non-harmful or beneficial manner, usually without having established a contractual relation beforehand’ (Thiede 2005, p. 1456). This mechanism is identified in DR2-1, DR2-2 and DR2-3, and implicit in DR1-8 and DR1-11. It shapes users’ perceptions of the health system, quality of care and providers’ professionalism (Gilson 2005; Goudge and Gilson 2005; Tibandebage and Mackintosh; 2005; Ostergaard 2015). Users’ trust in providers, based on their perceptions of those providers’ qualifications and professionalism and on past interactions, is thus linked with free public healthcare seeking. In fact, trust is built upon users’ expectations regarding providers’ technical skills, ability to communicate and listen, honesty and concern for patients’ well-being (Goudge and Gilson 2005).

Users’ awareness of risks associated with their health status was identified in DR2-4. This mechanism, which refers to users’ beliefs about the condition’s potential for harm, influences health behaviours, including healthcare seeking (Brewer *et al.* 2007). Andersen and Davidson (2007) named this social phenomenon ‘perceived need’ and defined it as:

… how people view their own general health and functional state. (…) Perceptions about the importance and magnitude of a health problem or symptom lead to a decision to seek medical care (or not to do so) (p. 7–8).

The Health Belief Model illustrates how these personal beliefs influence health behaviours. It identifies two types of perceptions—perceived seriousness of the condition and perceived susceptibility—that together influence perception of risk (Champion and Skinner 2008). These are notably influenced by information and advice to users from providers, which motivate healthcare seeking decisions (Thiede and McIntyre 2008). As the theoretical foundations of this mechanism are robust and empirical evidence is abundant in other contexts, we included it in the MRT, even though we identified it only once in the empirical literature.

Acceptability does not appear explicitly in the demi-regularities, but is manifested in several explanations of behaviours found in the empirical literature, especially as a sociocultural interpretation. This mechanism, which appears in DR1-9, DR1-10, DR1-11 and DR2-4, is closely linked to trust (Gilson 2007). Peters *et al.* (2008) define acceptability as ‘the match between how responsive providers are to the social and cultural expectations of individual users and communities’ (p. 162). This descriptive definition focuses on the health system and healthcare provision. From a realist perspective, we see acceptability as an explanatory concept in the healthcare seeking choice-making process. It refers to users’ perceptions of the acceptability of the choice to seek healthcare. It represents a process and evolves with users’ experience of healthcare. It is influenced not only by users’ sociocultural context, but also by social interactions that affect their social representations (Dillip *et al.* 2012). One example is a pregnant woman’s choice not to seek healthcare because the provider is a man. It could also refer to a resource allocation process, exemplified by a man’s choice not to seek healthcare for his under-5 daughter but to adopt a wait-and-see approach because the indirect costs of transportation would impair his ability to feed his family.

**Figure 4.**
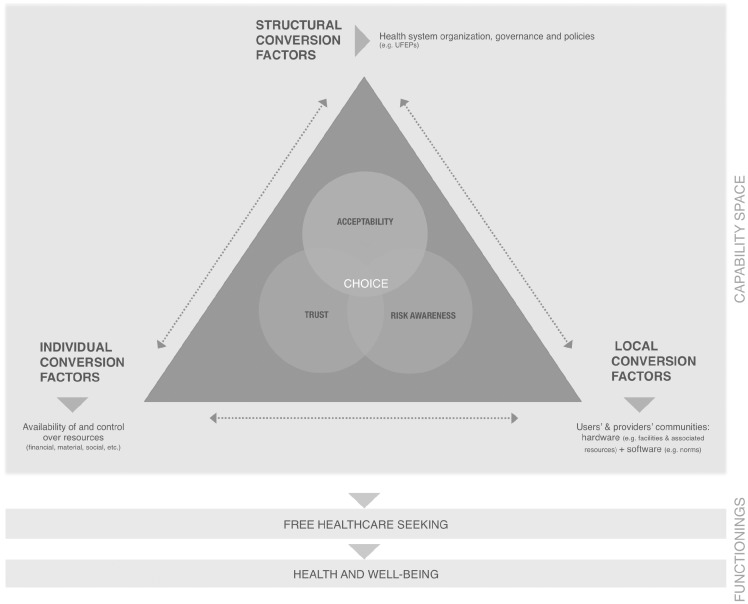
A realist MRT of free public healthcare seeking in SSA

We believe these three mechanisms have sufficient explanatory power to deepen our understanding of free healthcare seeking in various contexts, especially because they have been empirically tested and theorized elsewhere. The choice to seek free public healthcare is thus found at the intersection of three mechanisms: trust, risk awareness, and acceptability, as displayed in Figure 4.

According to Frediani (2010), people’s choice, ability, and opportunity to transform resources (e.g. free care) into functionings (e.g. seeking healthcare) make up the capability space. In Table 1, demi-regularities may relate to users’ choice or ability to act, because they reflect empirical findings. In the MRT, we offer a conceptual explanation of free healthcare seeking and the contextual elements that influence users’ capability space. That space is constrained or expanded by users’ context, conceptualized as individual, local and structural conversion factors. At the individual level, the availability of and control over resources is a compelling concept to explain the multiple interactions at play. It refers to users’ having not only the means to seek free care (e.g. financial resources for indirect costs), but also the opportunity to use those resources. An example would be a woman who needs to ask her husband’s permission to use household money to pay for transportation to the health facility. Resources are to be understood broadly, as including not only financial, but also material and social resources, knowledge, and information. This concept echoes that of capital, as defined by Obrist *et al.* (2007). Demi-regularities from the empirical literature highlight three key resources: financial resources (DR1-5, DR1-6, DR1-9), social resources (e.g. networks and status) (DR1-7, DR1-8, DR2-3), and knowledge and information (DR1-1, DR2-4). These offer individuals a social position, which influences their capability space. For example, indigents who experience humiliation or abuse at health facilities because of low social position may in turn distrust providers.

At the local level, Frediani (2010) defines conversion factors as facilities and collective norms. We conceptualized these factors as communities, each comprised of what Gilson (2012) calls ‘hardware’, including facilities and ‘software’, including norms. A community is:

- as suggested by MacQueen and colleagues, “A group of people with diverse characteristics who are linked by social ties, share common perspectives, and engage in joint action in geographical locations or settings” (MacQueen *et al.* 2001);
- venues or areas that are identified with key activities, such as residence, work, education, and recreation; and
- venues or areas that are physically-, geographically-, culturally-, and administratively- or geopolitically- defined.” (Goodman *et al.* 2014, p. S59)

Particularly influential in the healthcare seeking process were users’ and providers’ communities. Users belong to geographically defined communities characterized by the availability of facilities or resources, such as health facilities or transportation (DR1-3, DR1-4, DR1-5). They also belong to culturally defined communities, whose norms and values underlie the relationships and interactions among members (DR1-7, DR1-8) and with outsiders, such as providers (DR1-9, DR1-10, DR2-4). Providers (formal and informal) also belong to socially and physically defined communities, such as health facilities. Within health facilities, the hardware—which includes human resources, medical products, vaccines, technology etc.—influences users’ capability space (DR1-1, DR1-2, DR1-10). Meanwhile, social elements (software) forge the user-provider relationship, affecting users’ capability space (DR1-5, DR1-10, DR1-11, DR2-3). Interactions between software and hardware within providers’ communities are complex, shaping attitudes and behaviours toward users (DR2-2) and influencing users’ capability space. These interactions were not included in our review.

At the structural level, two conversion factors affect UFEP beneficiaries’ ability to choose to seek free public healthcare: health services being truly free (DR1-1, DR1-2), and users being informed of UFEP (DR1-1). These prerequisites show the distinction between a health policy’s values or principles and its modus operandi, in which activities and procedures (information strategy, financing mechanisms, monitoring etc.) must be implemented to achieve the intended outcomes. Several other contextual elements at the structural level (shown on Figure 4 under ‘Health system organization, governance and policies’) affect users’ capability space, including UFEPs. Other health interventions may also influence users’ capability space. For example, prenatal care programmes might increase women’s knowledge of pregnancy risk factors, triggering the mechanism of risk awareness and encouraging them to seek free care. Another example would be a human resources training intervention aimed at curbing providers’ shaming and abusive practices, triggering the mechanism of trust, and encouraging users’ choice to seek free care. In these examples, structural factors indirectly influence users’ capability space by influencing local factors. These relationships are shown as an arrow in Figure 4. Other structural conversion factors outside the health system, such as global health governance, non-health policies etc., were not found in the literature. We believe these warrant investigation, however, as some may substantially influence individual conversion factors and ultimately users’ capability space.

Arrows pointing to conversion factors show the interconnectedness of influences on users’ capability space. The examples above illustrate health policies’ influence on local conversion factors. Influences on individual conversion factors include demand-side policies, such as transportation loans or financial incentives (e.g. vouchers). Personal and local factors generally influence structural factors more diffusely and indirectly, and usually reflect feedback loops, particularly the reactions of actors affected by health system changes. Because these interactive and interdependent dimensions influence free healthcare seeking in complex and dynamic ways, illustrating them accurately with the necessary level of abstraction is challenging. This proposed MRT is an attempt to achieve this.

Its purpose is not to provide a detailed account of all existing or potential relationships between conversion factors, mechanisms and functionings. These are hypotheses that need to be tested and we encourage researchers to refine the MRT by deriving and testing such hypotheses, around such different diseases or health problems, and seeing whether such studies might prompt different CMO configurations. The proposed MRT is a generic explanation of the interactions between multiple elements that influence free public healthcare seeking in SSA.

## Discussion

### Contribution of the MRT to the study of healthcare access

Our aim in this realist review is to shed light on the contexts in which UFEPs stimulate greater use of public health services, and on the mechanisms they trigger. By making healthcare free at the point of delivery, UFEPs give users the opportunity to use public health services whenever they feel the need, without being dissuaded by cost. However, conversions factors influence users’ capability space, including their ability and choice to seize this opportunity. Our MRT offers a new conceptualization of free healthcare seeking in SSA based on realist principles, in which users’ choice to seek free care is influenced by trust, risk awareness and acceptability.

Research on healthcare access in LMICs is dominated by the study of determinants of health services use, the primary measure of access (Oliver and Mossialos 2004). The Five-As model of Penchansky and Thomas (1981), and Andersen’s (1995) behavioural model are the most widely used frameworks. Both have been adapted to LMICs (Obrist *et al.* 2007; Peters *et al.* 2008), but those adaptations are frameworks rather than theories. As such, they do not make the essential assumptions for a researcher be able ‘to diagnose a phenomenon, explain its processes and predict outcomes’ (Ostrom 1999, cited by Carpiano and Daley 2006, p. 565). Besides, their premises often focus on the supply/demand dichotomy. Knowledge syntheses have also been conducted on this theme (Dixon-Woods *et al.* 2006; Levesque *et al.* 2013), including several on LMICs (Thaddeus and Maine 1994; Gabrysch and Campbell 2009; Finlayson and Downe 2013; Cabieses and Bird 2014). Three were explicitly aimed at theory-building (Dixon-Woods *et al.* 2006; Finlayson and Downe 2013; Levesque *et al.* 2013), with two using a scientific approach to knowledge synthesis (Dixon-Woods *et al.* 2006; Finlayson and Downe 2013). Supplementary Material S1 provides a synopsis of existing theories, frameworks and models on access to healthcare and healthcare seeking, and assesses their relevance in relation to our theorizing process and focus. Our overview of healthcare access frameworks shows that, despite their contributions to knowledge advancement, most only list dimensions of access and some factors of use. None actually posits or explains how these interact with people and context to produce outcomes. As such, they offer a fragmented, rather than an integrative explanation. The healthcare-seeking process frameworks rarely consider theory on behaviour change or motivation and are thus atheoretical, besides suffering from the same limitations as the access frameworks. Moreover, no knowledge synthesis has been conducted on healthcare access in SSA with an explicit theory-building objective. Finally, existing works rarely examine the contexts within which users and providers interact or the interactions between those contexts and actors’ behaviours.

Our MRT proposes a configurational approach to formulating hypotheses and explaining outcomes. It seeks a holistic explanation that locates causation not in a statistical or temporal association between loosely defined factors or elements, and outcomes, but in a configurational association that links intervention to outcome by studying interactions between actors, contexts and mechanisms. Our MRT presents many advantages. It places users at the heart of the process, at a time when people-centered health systems are being promoted (Sheikh *et al.* 2014b). Because it incorporates the dynamic dimension of users’ experience, it differs from approaches based on the supply/demand (Ensor and Cooper 2004; Jacobs *et al.* 2012; Levesque *et al.* 2013). It also differs from pathways approaches (Thaddeus and Maine 1994; Currie and Wiesenberg 2003; Gabrysch and Campbell 2009; Levesque *et al.* 2013), while allowing for feedback loops that are inherent to the human experience and introduce a time dimension. Indeed, the mechanisms identified in our review take into account actors’ experience and reasoning, which are by definition always changing. Last, our MRT reinstates interpersonal values as the hub of this issue of healthcare access, relegating ‘hardware’ aspects (Sheikh *et al.* 2014a) to the periphery. Currently, most research in this field remains focused on ‘hardware’ at the expense of ‘software’, despite increased involvement of qualitative researchers and anthropologists in health policy and systems research (Gilson *et al.* 2011).

### Quality appraisal and limitations of the MRT

Our proposed MRT appears comprehensive enough to explain the healthcare seeking experience of different users for various conditions. However, it remains limited to UFEP beneficiaries’ experience and, as such, to the particular context of free healthcare in SSA. Our MRT thus meets the quality criterion of sensitivity to context (Whetten 1989). It also takes into account the multiplicity of issues, interactions, and contexts in the healthcare seeking process, illustrating the complexity of this phenomenon. It shows how the concepts are related, thereby satisfying the causality objective inherent to theory development (Whetten 1989). Finally, it draws on existing and robust theories and concepts and on empirical evidence analysed using the realist approach as the epistemology. Our approach thus enhances the credibility of our research (Whetten 1989).

A major limitation of our study is that stakeholders were rarely brought into the process (Robert 2015), whereas in realist reviews their participation is a measure of quality (Wong *et al.* 2013b). Thus, an interpretation bias is possible. To minimize this, the lead author regularly travelled to SSA to participate in conferences related to the research topic and to meet international, national and local UFEP stakeholders. Preliminary research results and successive interpretations were presented in various ways to diverse audiences, including researchers, health professionals, donor agencies and civil society representatives, to be enriched by their expert feedback. We also used peer review and debriefing processes, criteria for good quality research according to Cohen and Crabtree (2008), although these were not organized formally. The co-authors contributed actively to these processes, as did members of the University of Montreal’s West African UFEP student study group. Both have broad expertise on UFEPs and healthcare access in SSA. These approaches helped ensure the plausibility and accuracy of interpretations (Weick 1989; Irwin 2013).

Another limitation is the scope of our review. Our expectations for our research questions were not completely met. First, the issue of healthcare seeking is much broader than the results show; the number of mechanisms identified in our review was likely limited by the fact that our starting point was UFEPs in SSA. A realist review focusing on services use would provide a better understanding of interactions between these mechanisms and contextual factors. Such understanding might also have been enhanced by including other interventions aimed at providing free care. Second, time and resources constraints prevented us from fully investigating health workers’ role in UFEP implementation and its influence on users’ healthcare seeking process.

We thus paid a ‘price for incompleteness’ (Fein 2005), while nevertheless synthesizing a large body of evidence to provide an original perspective on free public healthcare seeking. This effort is part of the movement to produce and triangulate knowledge on healthcare access in LMICs, for which our MRT can serve as a starting point. We hope others will test and refine it accordingly.

### Lessons for realist reviews

Our research demonstrates the iterative and evolving nature of realist reviews. Starting with evaluating a public policy, we shifted to investigating a social phenomenon influenced by that policy. This shift was due to several factors, including our research interests and time spent on the review, conducted from 2010 to 2015. Through a process of trial and error, a thorough understanding of the phenomenon under study emerged through knowledge accretion. Our approach was akin to slow research (Adams *et al.* 2014), resisting anticipation, and thus far removed from rapid realist reviews (Saul *et al.* 2013). The result is an unexpected MRT on free healthcare seeking in SSA countries.

Our research process is close to analytic induction, a feature of qualitative research (Deslauriers 1997) that makes it, by definition, not reproducible. In light of this, quality assessment of this type of realist review should be based on standards that are specific. The RAMESES project has initiated such work and offers eight quality standards for realist syntheses (Wong *et al.* N/A) that include ‘constructing and refining a realist programme theory’ and ‘data extraction’. Although useful, those standards do not assess the theory-building process or the realist MRT. As a result, reviewers may be able to recognize what a good realist review is, but unable to discern a good realist MRT. Bourgeois’ (1979) work on middle-range theorizing and Weick’s (1989) and Whetten’s (1989) work on theory construction may be useful as starting points.

Finally, reporting the results of our realist review was a major challenge. We chose to present only the theory-building process and the final MRT, as we could not simultaneously report the evidence used to build the MRT in a single scientific article. Yet few articles give such a detailed description of these processes, despite the guidelines for publication of realist syntheses proposed by RAMESES (Wong *et al.* 2013a). Our choice is based on our commitment to transparency toward both researchers interested in realist reviews and audiences concerned about healthcare access in LMICs.

## Conclusion

To our knowledge, this study is the first to propose an MRT of free healthcare seeking in SSA using the realist approach. This theory rests on solid epistemological, theoretical and empirical foundations, as well as on a transparent research process. At a time when health policy and systems research in LMICs is taking shape (Gilson 2012), theory development is encouraged, as is the use of new research methods to convey the complexity of phenomena affecting health systems (Hercot *et al.* 2011; Gilson 2014). However, theory development remains the exception in this field. Articles dealing with theory development in a heuristic and reflexive way are particularly scarce. Thus, our goal was not only to share a new theoretical perspective on healthcare seeking, but also to ensure the transparency of the research process and to demonstrate the credibility of our results. Our aim has also been to show that both theory development and the realist approach are feasible and relevant to advance knowledge in the field of health policy and systems research.

## Supplementary Material

Supplement Figure 1

Supplement Data 1

Supplement Data 2
