# Supplementary figures and images for: Building a middle-range theory of free public healthcare seeking in sub-Saharan Africa: a realist review
Source: Health Policy Plan. 2017 May 16;32(7):1002–14. doi: 10.1093/heapol/czx035 (PMC5886156; doi:10.1093/heapol/czx035)

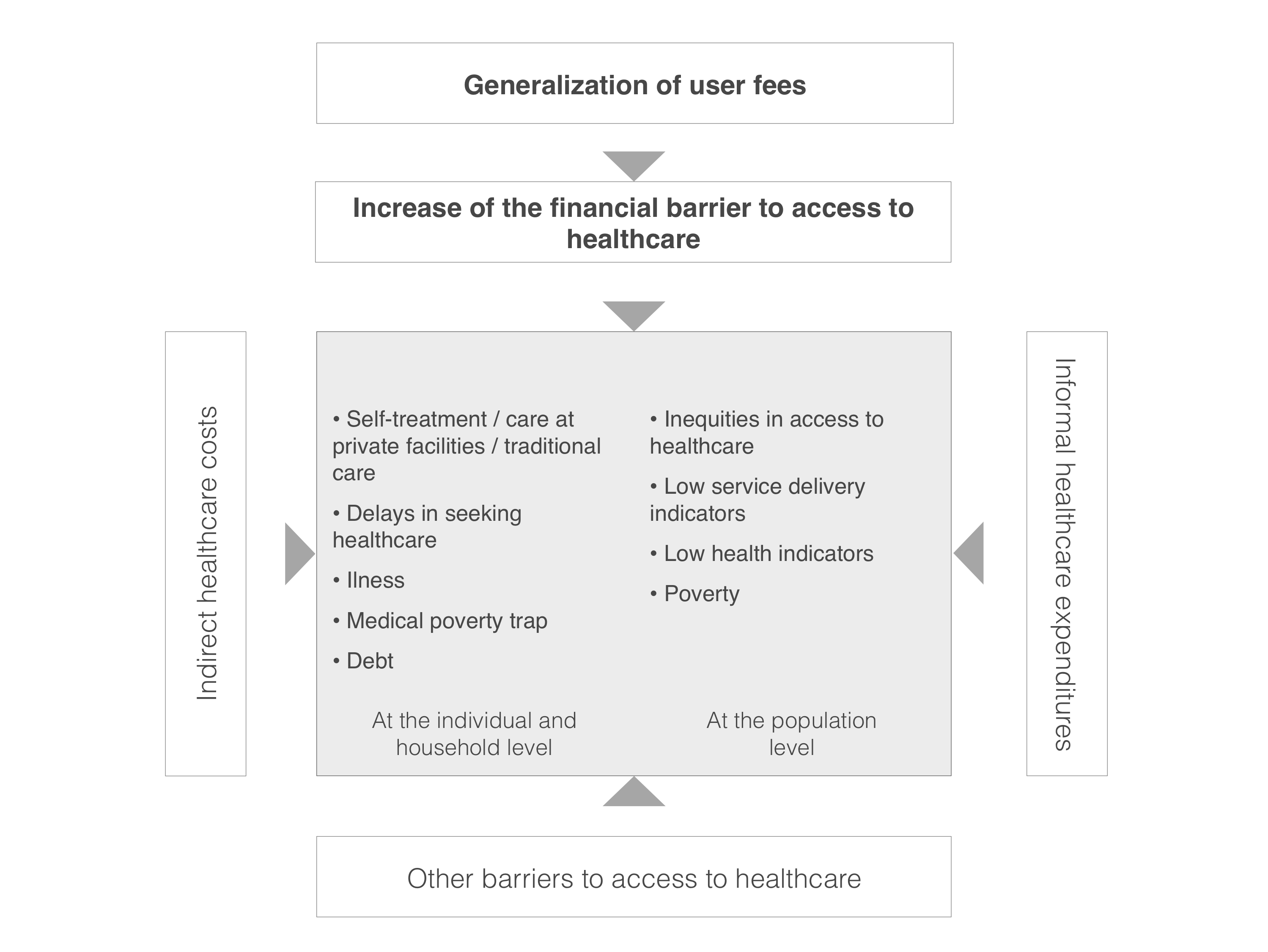

Supplement: Supplement Figure 1 [file supplementary_figure_1_final_czx035.png]
