# Supplementary material for: Building a middle-range theory of free public healthcare seeking in sub-Saharan Africa: a realist review
Source: Health Policy Plan. 2017 May 16;32(7):1002–14. doi: 10.1093/heapol/czx035 (PMC5886156; doi:10.1093/heapol/czx035)
Supplement: Supplement Data 1 [file supplementary_file_1_final_czx035.docx]

**Supplementary File 1 – Theories, frameworks and models on access to health care and healthcare seeking**

The following table summarizes the dimensions of the main theories, frameworks and models on access, use of health care and healthcare seeking. It is not exhaustive and presents, in chronological order, the main and the most recent writings, especially relating to low- and middle-income countries.

| **Year** | **Author(s)** | **Name (when applicable)** | **Dimensions** | **Main limits with regards to our theorizing process and focus** |
| --- | --- | --- | --- | --- |
| **Access to healthcare** | | | | |
| 1981 | **Penchansky, R., & Thomas, W. J.** | Five As (taxonomic definition of access) | Access is the “degree of “fit” between the clients and the system.   1. Availability (the relationship of the volume and type of existing services (and resources) to the clients' volume and types of needs) 2. Accessibility (the relationship between the location of supply and the location of clients) 3. Accommodation (the relationship between the manner in which the supply resources are organized to accept clients and the clients' ability to accommodate to these factors and the clients' perception of their appropriateness) 4. Affordability (the relationship of prices of services and providers' insurance or deposit requirements to the clients' income, ability to pay, and existing health insurance) 5. Acceptability (the relationship of clients' attitudes about personal and practice characteristics of providers to the actual characteristics of existing providers, as well as to provider attitudes about acceptable personal characteristics of clients) | - Taxonomy of dimensions (framework), rather than a theory - Focused on the measure of access, and on user’s satisfaction as the main outcome - Limited account of contextual influences on the dimensions of access - Provide a description of access at a certain point in time - Developed in the context of Western countries |
| 1995 | **Andersen, R.** | Behavioral model of health services use (4^th^ version) | 1. Environment (health care system / external environment) 2. Population characteristics (predisposing characteristics, enabling resources, needs) 3. Health behavior (personal health practices / use of health services) 4. Outcomes (perceived health status / evaluated health status / consumer satisfaction) | - Focused on the measure of access - Developed in the context of Western countries |
| 2003 | **Dixon-Woods, M., et al.** | Theoretical account of access to healthcare of vulnerable groups | “Candidacy describes how people's eligibility for healthcare is determined between themselves and health services. It is a continually negotiated property of individuals, subject to multiple influences arising both from people and their social contexts and from macro-level influences on allocation of resources and configuration of services. Health services are continually constituting and seeking to define the appropriate objects of medical attention and intervention, while at the same time people are engaged in constituting and defining what they understand to be the appropriate objects of medical attention and intervention. Access represents a dynamic interplay between these simultaneous, iterative and mutually reinforcing processes.”  Dimensions: candidacy / navigation / permeability of services / appearances at health services / adjudications / offers and resistance | - Definition of constructs, rather than a theory - Focused on “receipt” of healthcare, in the UK context - Limited integration of contextual influences in theorization |
| 2007 | **Obrist, B., et al.** | Health Access Livelihood Framework | “Five dimensions of access influence the course of the health-seeking process: Availability, Accessibility, Affordability, Adequacy, and Acceptability. What degree of access is reached along the five dimensions depends on the interplay between (a) the health care services and the broader policies, institutions, organizations, and processes that govern the services, and (b) the livelihood assets people can mobilize in particular vulnerability contexts. However, improved access and health care utilization have to be combined with high quality of care to reach positive outcomes. The outcomes can be measured in terms of health status (as evaluated by patients or by experts), patient satisfaction, and equity.”   1. Outcomes (health status, patient satisfaction, equity) 2. Five As (availability, accessibility, affordability, adequacy and acceptability) 3. Influenced by :    - Healthcare services (health facilities, private practice, drug shops, traditional healers and others) influenced by the broader context of policies, institutions, organizations and processes    - Livelihood assets (physical, human, financial, natural and social capitals) influenced by the vulnerability context of households | - Framework, rather than a theory - Conflation of healthcare seeking process and dimensions of access to healthcare |
| 2008 | **Peters, D. H., et al.** | Conceptual framework for assessing access to health services in developing countries | Access is related to the timely use of services according to need.   1. Geographic accessibility—the physical distance or travel time from service delivery point to the user 2. Availability—having the right type of care avail- able to those who need it, such as hours of operation and waiting times that meet demands of those who would use care, as well as having the appropriate type of service providers and materials 3. Financial accessibility—the relationship between the price of services (in part affected by their costs) and the willingness and ability of users to pay for those services, as well as be protected from the economic consequences of health costs 4. Acceptability—the match between how responsive health service providers are to the social and cultural expectations of individual users and communities | - Framework, rather than a theory - Quality of care at the centre of the framework |
| 2009 | **McIntyre, D., Thiede, M., & Birch, S.** | Access as empowerment (access evaluation framework) | 1. Availability: whether the appropriate health care providers or services are supplied in the right place and at the right time to meet the prevailing needs of the population 2. Affordability : ‘degree of fit’ between the full costs to the individual of using the service and the individual’s ability to pay in the context of the household budget and other demands on that budget 3. Acceptability : fit between provider and patient attitudes towards and expectations of each other | - Framework, rather than a theory - Limited integration of contextual influences |
| 2010 | **Rutherford, M., Mulholland, K., & Hill, P.** | Access to health care in sub-Saharan Africa impacting under-5 mortality | 1. Traditionally measured variables: distance to a health provider and cost of obtaining health care)   2) Additional variables (social support, time availability and caregiver autonomy) | - Synthesis of measures of access variables which impact under-five mortality in sub-Saharan Africa, rather than a theory |
| 2011 | **Carrillo, J. E., et al.** | Health Care Access Barriers Model (HCAB) | “The HCAB model focuses on those health care access barriers that are associated with adverse health care circumstances leading to health disparities.”   1. Health care access barriers: Financial / Cognitive / Structural barriers 2. Outcomes : Late presentation / Decrease prevention / Decrease care 3. Health outcomes disparities | - Focused on health care access barriers that can be modified, as a way to help design community intervention - Framework, rather than a theory |
| 2013 | **Levesque, J. F., Harris, M. F., & Russell, G.** | Patient-centred conceptual framework of access to health care | “access is defined as the opportunity to reach and obtain appropriate health care services in situations of perceived need for care”   - Accessibility of providers, organisations, institutions, systems - Ability of populations, communities, households, individuals  1. Outcomes as a process (perception of needs and desire for care / health care seeking / health care reaching / health care utilisation / health care consequences) 2. Supply-side determinants:    - Approachability (transparency, outreach, information, screening)    - Acceptability (professional values, norms, culture, gender)    - Availability and accommodation (geographic location, accommodation, hours of opening, appointments mechanisms)    - Affordability (direct costs, indirects costs, opportunity costs)    - Appropriateness (technical and interpersonal quality, adequacy, coordination and continuity) 3. Demand-side determinants :    - Ability to perceive (heath literacy, health beliefs, trust and expectations)    - Ability to seek (personal and social values, culture, gender, autonomy)    - Ability to reach (living environments, transport, mobility, social support)    - Ability to pay (income, assets, social capital, health insurance)    - Ability to engage (empowerment, information, adherence, caregiver support) | - No explicit theorization process - Limited integration of contextual influences (outside individual ability and health system characteristics) |
| 2014 | **Cabieses, B., &**  **Bird, P.** | Glossary + framework of access to health care for LMICs | 1. Context 2. Need for health care 3. Access to health care (access, barriers to access, and related concepts) 4. Utilization of health care 5. Goals in health care | - Framework to organize the glossary, rather than a theory - No integration of contextual influences - Limited explanation of interactions of dimensions and variables |
| **Healthcare seeking process** | | | | |
| 1994 | **Thaddeus, S., & Maine, D.** | Three delays model | 1. Delay 1: recognizing danger signs and deciding to seek care    - Low status of women    - Poor understanding of complications and risk factors in pregnancy and of when medical interventions are needed    - Previous poor experience of health care    - Acceptance of maternal death    - Financial implications 2. Delay 2: reaching appropriate care    - Distance to health centres and hospitals    - Availability of and cost of transportation    - Poor roads    - Geography e.g. mountainous terrain, rivers 3. Delay 3: receiving quality care at health facilities    - Poor facilities and lack of medical supplies    - Inadequately trained and poorly motivated medical staff    - Inadequate referral systems | - Focused on maternal mortality and obstetrical complications - Expressed in terms of obstacles to healthcare seeking and receiving - Healthcare seeking decision-making conceptualized as one step of the process - Process model with healthcare seeking decision-making separated from ability to access health services |
| 2003 | **Currie, D., & Wiesenberg, S.** | Women’s health-seeking behavior as decision-making | 1. Do I have a health problem? (knowledge, threshold of illness) 2. Can I seek healthcare? (authority in the household, financial resources, social value of women’s (lost) time, mobility of women) 3. Will I seek healthcare? (accessibility, gender and maturity of health practitioners, anticipated quality of care, attitudes toward the female body) | - Tool for action, rather than a theory - Focused on women and sexual and reproductive health |
| 2009 | **Gabrysch, S., & Campbell, O. M.** | Delay phases and factors affecting use of delivery care and maternal mortality in LMICs | Based on Thaddheus and Maine “three delays” model, adding preventive care-seeking.   1. Phases :    - Phase 1 : Deciding to seek preventive care for delivery / deciding to seek care for complication    - Phase 2 : Identifying and reaching health facility    - Phase 3 : Receiving normal delivery care at health facility / receiving adequate and appropriate treatment for complication 2. Factors affecting use of delivery care    - Sociocultural factors (phase 1)    - Perceived benefit / need (phase 1)    - Economic accessibility (phase 1 and 2)    - Physical accessibility (phase 1 and 2)    - Quality of care (phase 1 and 3) | - No explicit theorization process - Focused on maternal health - Healthcare seeking decision-making conceptualized as one step of the process |
| 2013 | **Finlayson, K., & Downe, S.** | Explanatory model of use of antenatal services in LMICs | 1. Pregnancy as socially contingent and physiologically healthy    - Pregnancy awareness and disclosure    - Resistance to risk-averse care models 2. Resource use and survival in conditions of extreme poverty    - Using resources for health care or basic survival    - Difficult and dangerous travel 3. Not getting it right the first time    - Attending clinics is not worth the effort    - Locally determined rules of access    - Disrespect and abuse | - Focused on antenatal services - Limited integration of contextual influences |

**References**

1. Andersen, R. (1995). Revisiting the behavioral model and access to medical care: Does it matter? *Journal of Health and social behaviour, 36*, 1-10.
2. Cabieses, B., & Bird, P. (2014). Glossary of access to health care and related concepts for low- and middle-income countries (LMICs): a critical review of international literature. *Int J Health Serv, 44*(4), 845-861.
3. Carrillo, J. E., Carrillo, V. A., Perez, H. R., Salas-Lopez, D., Natale-Pereira, A., & Byron, A. T. (2011). Defining and targeting health care access barriers. *J Health Care Poor Underserved, 22*(2), 562-575. doi: 10.1353/hpu.2011.0037
4. Currie, D., & Wiesenberg, S. (2003). Promoting women's health-seeking behavior: Research and the empowerment of women. *Health Care Women Int, 24*(10), 880-899. doi: 10.1080/07399330390244257
5. Dixon-Woods, M., Cavers, D., Agarwal, S., Annandale, E., Arthur, A., Harvey, J., . . . Sutton, A. J. (2006). Conducting a critical interpretive synthesis of the literature on access to healthcare by vulnerable groups. *BMC Med Res Methodol, 6*, 35. doi: 10.1186/1471-2288-6-35
6. Finlayson, K., & Downe, S. (2013). Why do women not use antenatal services in low- and middle-income countries? A meta-synthesis of qualitative studies. *PLoS Med, 10*(1), e1001373. doi: 10.1371/journal.pmed.1001373
7. Gabrysch, S., & Campbell, O. M. (2009). Still too far to walk: Literature review of the determinants of delivery service use. *BMC Pregnancy Childbirth, 9*, 34. doi: 10.1186/1471-2393-9-34
8. Levesque, J. F., Harris, M. F., & Russell, G. (2013). Patient-centred access to health care: Conceptualising access at the interface of health systems and populations. *Int J Equity Health, 12*, 18. doi: 10.1186/1475-9276-12-18
9. McIntyre, D., Thiede, M., & Birch, S. (2009). Access as a policy-relevant concept in low- and middle-income countries. *Health Econ Policy Law, 4*(Pt 2), 179-193. doi: 10.1017/S1744133109004836
10. Obrist, B., Iteba, N., Lengeler, C., Makemba, A., Mshana, C., Nathan, R., . . . Mshinda, H. (2007). Access to health care in contexts of livelihood insecurity: A framework for analysis and action. *PLoS Med, 4*(10), e308. doi: 10.1371/journal.pmed.0040308
11. Penchansky, R., & Thomas, W. J. (1981). The concept of access: Definition and relationship to consumer satisfaction. *Medical Care, 19*(2), 127-140.
12. Peters, D. H., Garg, A., Bloom, G., Walker, D. G., Brieger, W. R., & Rahman, M. H. (2008). Poverty and access to health care in developing countries. *Ann N Y Acad Sci, 1136*, 161-171. doi: 10.1196/annals.1425.011
13. Rutherford, M., Mulholland, K., & Hill, P. (2010). How access to health care relates to under-five mortality in sub-Saharan Africa: Systematic review. *Tropical medicine & international health, 15*(5), 508-519.
14. Thaddeus, S., & Maine, D. (1994). Too far to walk: maternal mortality in context. *Soc Sci Med, 38*(8), 1091-1110.
