# Supplementary material for: Building a middle-range theory of free public healthcare seeking in sub-Saharan Africa: a realist review
Source: Health Policy Plan. 2017 May 16;32(7):1002–14. doi: 10.1093/heapol/czx035 (PMC5886156; doi:10.1093/heapol/czx035)
Supplement: Supplement Data 2 [file supplementary_file_2_final_czx035.doc]

**Supplementary File 2. List of empirical studies included in the realist review**

| **Burundi** |
| --- |
| Nimpagaritse, M. & Bertone, M. P. 2011. The sudden removal of user fees: The perspective of a frontline manager in Burundi. *Health Policy and Planning,* 26: ii63-ii71. |
| Observatoire de l'Action Gouvernementale 2009. *Évaluation des effets de la mesure de subvention des soins pour les enfants de moins de 5 ans et pour les accouchements sur les structures et la qualité des soins,* Bujumbura, Burundi: OAG, 74. |
| **Ghana** |
| Agyepong, I. A. & Nagai, R. A. 2010. "We charge them; otherwise we cannot run the hospital": Front line workers, clients and health financing policy implementation gaps in Ghana. *Health Policy,* 99: 226-233. |
| Dzakpasu, S., Soremekun, S., Manu, A., et al. 2012. Impact of free delivery care on health facility delivery and insurance coverage in Ghana's Brong Ahafo Region. *PLoS One,* 7: e49430. |
| Grepin, K. A. 2009. *Influencing health systems: Priorities, policies, and providers,* Cambridge, MA: Harvard University, 85. |
| Mills, S., Williams, J. E., Adjuik, M. & Hodgson, A. 2008. Use of health professionals for delivery following the availability of free obstetric care in northern Ghana. *Maternal and Child Health Journal,* 12: 509-18. |
| Penfold, S., Harrison, E., Bell, J. & Fitzmaurice, A. 2007. Evaluation of the delivery fee exemption policy in Ghana: Population estimates of changes in delivery service utilization in two regions. *Ghana Medical Journal,* 41: 100-9. |

| Witter, S. & Adjei, S. 2007. Start-stop funding, its causes and consequences: A case study of the delivery exemptions policy in Ghana. *International Journal of Health Planning and Management,* 22: 133-43. |
| --- |
| Witter, S., Kusi, A. & Aikins, M. 2007. Working practices and incomes of health workers: evidence from an evaluation of a delivery fee exemption scheme in Ghana. *Human Resources for Health,* 5: 2-2. |
| Witter, S., Arhinful, D. K., Kusi, A. & Zakariah-Akoto, S. 2007. The experience of Ghana in implementing a user fee exemption policy to provide free delivery care. *Reproductive Health Matters,* 15: 61-71. |
| Witter, S., Kusi, A. & Aikins, M. 2007. Working practices and incomes of health workers: evidence from an evaluation of a delivery fee exemption scheme in Ghana. *Human Resources for Health,* 5: 2-2. |
| **Madagascar** |
| Fafchamps, M. & Minten, B. 2006. Public service provision, user fees and political turmoil. *Journal of African Economies,* 16: 485-518. |
| **Niger** |
| Diarra, A. 2011. *La mise en œuvre de la politique d'exemption de paiements dans les districts sanitaires de Gaweye et de Say,* Niamey, Niger: LASDEL, 67. |
| Diarra, A. 2012. Mise en œuvre locale de l'exemption des paiements des soins au Niger. *Afrique Contemporaine,* 243: 77-93. |
| Diarra, A. 2011. *La mise en œuvre de la politique d'exemption de paiements dans les districts sanitaires de Gaweye et de Say,* Niamey, Niger: LASDEL, 67. |

| Ousseini, A. 2011. *Une politique publique de santé au Niger: La mise en place d'exemptions de paiement des soins en faveur des femmes et des enfants,* Niamey, Niger: LASDEL, 60. |
| --- |
| Page, A. L. & Hustache, S. 2011. Health care seeking behavior for diarrhea in children under 5 in rural Niger: Results of a cross-sectional survey. *BMC Public Health,* 11: 389. |
| Ridde, V., Diarra, A. & Moha, M. 2011. User fees abolition policy in Niger: Comparing the under five years exemption implementation in two districts. *Health Policy,* 99: 219-225. |
| **Senegal** |
| Witter, S., Drame, F. & Cross, S. 2009. Maternal fee exemption in Senegal: Is the policy a success? *African Journal of Midwifery and Women's Health,* 3: 5-10. |
| Witter, S. & Diadhiou, M. 2008. Key informant views of a free delivery and caesarean policy in Senegal. *African Journal of Reproductive Health,* 12: 93-111. |
| Witter, S., Dieng, T., Mbengue, D., et al. 2010. The national free delivery and caesarean policy in Senegal: Evaluating process and outcomes. *Health Policy and Planning,* 25: 384-92. |
| Ministère de la santé et de la prévention médicale 2007. *Évaluation des stratégies de réduction des barrières économiques, socioculturelles, sanitaires et institutionnelles à l'accès aux soins obstétricaux et néonataux au Sénégal,* Dakar, Sénégal: Ministère de la santé et de la prévention médicale, Ministère de la santé et de la prévention médicale, Fonds des Nations Unies pour la Population, Initiative for maternal mortality programme assessment, Centre de formation et de recherche en santé de la reproduction, 74. |

| **Sierra Leone** |
| --- |
| Amnesty International 2011. *At a crossroads: Sierra Leone's free health care policy,* London, UK: Amnesty International, 41. |
| Diaz, T., George, A. S., Rao, S. R., et al. 2013. Healthcare seeking for diarrhoea, malaria and pneumonia among children in four poor rural districts in Sierra Leone in the context of free health care: Results of a cross-sectional survey. *BMC Public Health,* 13: 157. |
| **South Africa** |
| Bhayat, A. & Cleaton-Jones, P. 2003. Dental clinic attendance in Soweto, South Africa, before and after the introduction of free primary dental health services. *Community Dentistry and Oral Epidemiology,* 31: 105-10. |
| Brink, A. S. & Koch, S. F. 2013. *The 1996 user fee abolition in South Africa: A difference-in-difference analysis,* Pretoria, South Africa: University of Pretoria, 23. |
| Goudge, J., Gilson, L., Russell, S., et al. 2009. The household costs of health care in rural South Africa with free public primary care and hospital exemptions for the poor. *Tropical Medicine and International Health,* 14: 458-67. |
| Goudge, J., Russell, S., Gilson, L., et al. 2009. Illness-related impoverishment in rural South Africa: Why does social protection work for some households but not others? *Journal of International Development,* 21: 231-251. |
| Knight, L. & Maharaj, P. 2009. Use of public and private health services in KwaZulu-Natal, South Africa. *Development Southern Africa,* 26: 17-28. |
| Netshandama, V. O., Nemathaga, L. & Shai-Mahoko, S. N. 2005. Experiences of primary health care nurses regarding the provision of free health care services in the northern region of the Limpopo Province. *Curationis,* 28: 59-68. |
| Power, M., Eis, R., Zwarenstein, M., et al. 1997. Most patients attending a 'walk-in' clinic at Red Cross War Memorial Children's Hospital could safely be managed at primary care level. *South African Medical Journal,* 87: 36-41. |
| Walker, L. & Gilson, L. 2004. 'We are bitter but we are satisfied': Nurses as street-level bureaucrats in South Africa. Social Science & Medicine, 59: 1251-61. |
| Wilkinson, D., Sach, M. E., Abdool Karim, S. S. & Costello, A. 1997. Examination of attendance patterns before and after introduction of South Africa's policy of free health care for children aged under 6 years and pregnant women. British Medical Journal, 314: 940-941. |
| Wilkinson, D., Gouws, E., Sach, M. & Karim, S. S. 2001. Effect of removing user fees on attendance for curative and preventive primary health care services in rural South Africa. Bulletin of the World Health Organization, 79: 665-71. |
| **Tanzania** |
| Ferry, G., Dickson, S. R., Mbaruku, G., et al. 2012. Equity of inpatient health care in rural Tanzania: A population- and facility-based survey. International Journal for Equity in Health, 11: 7. |
| Kahabuka, C., Kvåle, G., Moland, K. M. & Hinderaker, S. G. 2011. Why caretakers bypass primary health care facilities for child care: A case from rural Tanzania. BMC Health Services Research, 11: 315. |
| Kahabuka, C., Moland, K. M., Kvåle, G. & Hinderaker, S. G. 2012. Unfulfilled expectations to services offered at primary health care facilities: Experiences of caretakers of underfive children in rural Tanzania. BMC Health Services Research, 12: 158. |
| Kruk, M. E., Mbaruku, G., McCord, C. W., et al. 2009. Bypassing primary care facilities for childbirth: A population-based study in rural Tanzania. Health Policy and Planning, 24: 279-88. |

| Kruk, M. E., Mbaruku, G., Rockers, P. C. & Galea, S. 2008. User fee exemptions are not enough: Out-of-pocket payments for 'free' delivery services in rural Tanzania. *Tropical Medicine and International Health,* 13: 1442-51. |
| --- |
| Kruk, M., Paczkowski, M., Mbaruku, G., et al. 2009. Women's preferences for place of delivery in rural Tanzania: A population-based discrete choice experiment. *American Journal of Public Health,* 99: 1666-72. |
| Kruk, M. E., Rockers, P. C., Mbaruku, G., et al. 2010. Community and health system factors associated with facility delivery in rural Tanzania: A multilevel analysis. *Health Policy,* 97: 209-16. |
| Magoma, M., Requejo, J., Campbell, O. M. R., et al. 2010. High ANC coverage and low skilled attendance in a rural Tanzanian district: A case for implementing a birth plan intervention. *BMC Pregnancy and Childbirth,* 10: 13. |
| Manzi, F., Schellenberg, J. A., Adam, T., et al. 2005. Out-of-pocket payments for under-five health care in rural southern Tanzania. *Health Policy and Planning,* 20 Suppl 1: i85-i93. |
| Mpembeni, R. N., Killewo, J. Z., Leshabari, M. T., et al. 2007. Use pattern of maternal health services and determinants of skilled care during delivery in Southern Tanzania: Implications for achievement of MDG-5 targets. *BMC Pregnancy and Childbirth,* 7: 29-29. |
| Mrisho, M., Schellenberg, J. A., Mushi, A. K., et al. 2007. Factors affecting home delivery in rural Tanzania. *Tropical Medicine and International Health,* 12: 862-72. |
| Perkins, M., Brazier, E., Themmen, E., et al. 2009. Out-of-pocket costs for facility-based maternity care in three African countries. *Health Policy and Planning,* 24: 289-300. |

| Pfeiffer, C. & Mwaipopo, R. 2013. Delivering at home or in a health facility? Health-seeking behaviour of women and the role of traditional birth attendants in Tanzania. *BMC Pregnancy and Childbirth,* 13: 55. |
| --- |
| **Uganda** |
| Deininger, K. & Mpuga, P. 2005. Economic and welfare impact of the abolition of health user fees: Evidence from Uganda. *Journal of African Economies,* 14: 55-91. |
| Kajula, P., Kintu, F., Barugahare, J. & Neema, S. 2004. Political analysis of rapid change in Uganda's health financing policy and consequences on service delivery for malaria control. *International Journal of Health Planning and Management,* 19: S133-53. |
| Kiguli, J., Ekirapa-Kiracho, E., Okui, O., et al. 2009. Increasing access to quality health care for the poor: Community perceptions on quality care in Uganda. *Patient Preference and Adherence,* 3: 77-85. |
| Mpuga, P. 2002. *Health outcomes after the abolition of Cost-sharing in public hospitals in Uganda,* Linz, Austria: Johannes Kepler University, 50. |
| Nabyonga, J., Desmet, M., Karamagi, H., et al. 2005. Abolition of cost-sharing is pro-poor: Evidence from Uganda. *Health Policy and Planning,* 20: 100-8. |
| Nabyonga-Orem, J., Karamagi, H., Atuyambe, L., et al. 2008. Maintaining quality of health services after abolition of user fees: A Uganda case study. *BMC Health Services Research,* 8: 102-102. |
| Nabyonga-Orem, J., Mugisha, F., Kirunga, C., et al. 2011. Abolition of user fees: The Uganda paradox. *Health Policy and Planning,* 26: ii41-ii51. |
| Pariyo, G., Ekirapa-Kiracho, E., Okui, O., et al. 2009. Changes in utilization of health services among poor and rural residents in Uganda: Are reforms benefitting the poor? *International Journal for Equity in Health,* 8: 39-39. |
| Rutebemberwa, E., Pariyo, G., Peterson, S., et al. 2009. Utilization of public or private health care providers by febrile children after user fee removal in Uganda. *Malaria Journal,* 8: 45. |
| Rutebemberwa, E., Kallander, K., Tomson, G., et al. 2009. Determinants of delay in care-seeking for febrile children in eastern Uganda. *Tropical Medicine and International Health,* 14: 472-9. |
| Twikirize, J. M. & O'Brien, C. 2012. Why Ugandan rural households are opting to pay community health insurance rather than use the free healthcare services. *International Journal of Social Welfare,* 21: 66-78. |
| Xu, K., Evans, D. B., Kadama, P. Y., et al. 2006. Understanding the impact of eliminating user fees: Utilization and catastrophic health expenditures in Uganda. *Social Science and Medicine,* 62: 866-76. |
| **Zambia** |
| Chama-Chiliba, C. & Koch, S. 2013. *Evaluating the impact of the abolition of user fees on facility-based deliveries in rural Zambia,* Oxford, UK: Centre for the Study of African Economies Conference, 31. |
| Carasso, B., Lagarde, M., Cheelo, C., et al. 2012. Health worker perspectives on user fee removal in Zambia. *Human Resources for Health,* 10: 40. |
| Lagarde, M. 2010. *Analysis of the impact of user fee removal on health seeking behaviours using a difference-in-differences approach,* Harare, Zambia: Ministry of Health Zambia / University of Zambia / London School of Hygiene & Tropical Medicine / University of Cape Town, 37. |
| Lagarde, M. & Palmer, N. 2010. *Removal of user fees in Zambia: Was the impact sustained over time?,* Harare, Zambia: Ministry of Health Zambia / University of Zambia / London School of Hygiene & Tropical Medicine / University of Cape Town, 27. |
| Ngulube, T. J. & Carasso, B. 2010. *Removal of user fees in Zambia: What has happened in communities?,* Harare, Zambia: Ministry of Health Zambia / University of Zambia / London School of Hygiene & Tropical Medicine / University of Cape Town, 44. |
| Hadley, M. 2011. Does increase in utilisation rates alone indicate the success of a user fee removal policy? A qualitative case study from Zambia. *Health Policy,* 103: 244-254. |
| Lagarde, M., Barroy, H. & Palmer, N. 2012. Assessing the effects of removing user fees in Zambia and Niger. *Journal of Health Services Research and Policy,* 17: 30-6. |
| Masieye, F., Chitah, B. M., Chanda, P. & Simeo, F. 2008. *Removal of user fees at Primary Health Care facilities in Zambia: A study of the effects on utilisation and quality of care,* Harare, Zambia: Regional Network for Equity in Health in east and southern Africa (EQUINET) / University of Cape Town, 21. |
| Masiye, F., Chitah, B. M. & McIntyre, D. 2010. From targeted exemptions to user fee abolition in health care: Experience from rural Zambia. *Social Science and Medicine,* 71: 743-50. |
| *Note : Some studies deal with more than one country included in the review.* |
